# Annotations

**Published:** 1900-06-23

**Authors:** 


### Annotations.

### War and its Wounded.

The tendency of man, and also of nations, is to become self-centred and to regard his own, or its own, affairs as if they were the greatest and most important events of the era. And never does this lack of perspective, this inability to recognise things in their due proportion show itself with greater force than when man fights his battles o'er again, and nations hug themselves with jubilation about success in war. What a wonderful war! What gallantry of troops! What splendid medical arrangements! What bravery among doctors and stretcher bearers! What hospitals! What ambulance trains! What difficulties for British pluck to struggle against and overcome; and what brave fellows we all are! But if we peep through the glasses of experience, and regard the matter in the perspective of history, things take more simple proportions, and our doings tend perhaps to occupy not quite so large a share of the foreground. Amid all that is being said about the super-excellence of our medical arrangements it may be well perhaps to hint that, great as is the force at present engaged in South Africa, it is scattered over a vast extent of country, that in no one battle has a really large army been engaged, and that so far as the number of wounded thrown upon the hands of the medical department is concerned it has been quite trifling in comparison with what has happened in great Continental wars, and what will happen again, perhaps even at our doors, which God forfend, should the great nations ever be driven to engage in mortal combat. On the occasion of being presented with the freedom of the Salters' Company last Monday, Sir William MacCormac—who speaks with experience about these matters—drew comparisons between the losses in the present war and those in the Franco-Prussian war in 1870. Our losses, he said, were very grievous, but when we thought of Saint Privat, where 8,000 of the choicest Prussian Guards were laid low in 11 minutes; and of Sedan, where alone 10,000 French were killed and wounded, our figures changed into insignificance. While, then, we are thankful that our wounded have been well looked after, as, indeed, they have been, and that our losses have kept within moderate dimensions, do not let us imagine that our medical arrangements have passed their examination and have come out with honours. The fact is that no real test has been made in the sense in which tests are understood among those who speak of great wars, and that the medical department will have to share in that general overhauling of all army affairs which, we hope, is inevitable so soon as this war is safely over. After all, some lessons of the war are fairly obvious, and among those which are beginning to stand out most forcibly is the necessity of utilising the non-professional. For a long series of years our army has been mainly engaged in what may, without offence, we hope, be termed matters of police, and its organisation has been made on that basis. But when great wars are in question that form of organisation will probably turn out the best which is most effectual in bringing the whole power of the nation to bear upon the matter in hand. The control and the policing of our great empire is a big business, and will not only furnish occupation for, but will also provide an admirable training school for a large army of professional soldiers. But whenever it comes to a matter of real war this is not the army which will have to do the fighting. Engineers and artillery, generals and drill sergeants, and, above all, a complete organisation must be professional; but the men, the subordinate officers, the transport, the commissariat, the medical department, the nursing, the ambulance arrangements, will all have to be provided on so vast a scale that resource must of necessity be had to those who in times of peace are mainly occupied in the peaceful work of earning bread and butter. To make the nation a fighting force, organisation on quite a new scale will be found essential. "Little wars," frontier fights, punitive expeditions, and so forth—what we may call police wars—will be carried out by our professional soldiers. But when it becomes a question of nation against nation the nation itself must fight, and, in view of this possibility, we must accept the inevitable. No possible army medical department could bear the strain if unassisted, and the provision of this assistance should be part of its organisation. So far as our side of the question is concerned, we have no doubt that not only will it become necessary to largely increase the numerical efficiency of the Royal Army Medical Corps, but it will be found essential so to arrange matters that it shall always have at its back, as part of its recognised organisation, a large number of young, active medical men, nurses, stretcher-bearers, and orderlies, normally engaged in civil practice but well trained in military duties, and ready at any moment to fall into their proper places so soon as the army corps to which they are attached is mobilised.

### Fowls and Diphtheria.

A most interesting question in regard to some of the infectious maladies from which man suffers is the extent to which they also affect the animals by which man is constantly surrounded. Partly for the sake of their services and partly for the sake of the food which they supply, man has in every country surrounded himself and his habitations with domestic animals of many kinds; while other animals in turn, finding man and his waste products useful to them, have taken to living a parasitic life upon and around him and his dwellings; and when attempts are made to suppress epidemics by isolating those who are affected, it is obviously a matter of the first importance to ascertain the extent to which man's associates are liable to these diseases and are capable of spreading the infection. Markedly is this the case with tuberculosis. We all know about the rats and the way they spread plague. The influence of the

ANNOTATIONS—*continued.*

domestic fly in spreading typhoid fever now is well recognised, while the relation between the domestic dog and hydrophobia has long been commonplace. How far the domestic fowl is liable to diphtheria is a question which is still *sub judice*. That cats sometimes suffer from it and are responsible for its introduction into families seems undoubted, but there is much reason for believing that fowls also may become affected, although they may not be so dangerous a source of infection. There has been much discussion lately as to how far the malady known as "gapes" in birds is or is not diphtheria. The fact would seem to be that the word has been very loosely used, and that although when properly applied it means a definite disease caused by a nematode worm, it is often employed, as is also the word "roup," to indicate an epidemic disorder which is asserted by some to be diphtheria. This disorder is stated to run concurrently with human diphtheria, and, what is of considerable importance, it is stated that after an attack birds are apt to suffer from a form of paralysis analogous to that which follows diphtheria in humans. If one merely considers what are called probabilities, it certainly would seem in accordance with known facts that domestic animals, so intimately associated with man and his food as is the domestic fowl, should share with him a disease like diphtheria.

**Working-Class Prosperity.**

Let us rejoice with our brother the working man, for shall not his prosperity mean joy and gladness—nay, even fulness of the money-bags and peace of mind—to many a hard pressed medical practitioner? The *Labour Gazette* for this month shows the enormous increase which has taken place in the wages of large groups of the working classes during the last five months; and when we consider how large a proportion of the work of doctors is done among weekly wage earners it cannot be without interest to note this turn of affairs, for if now is the time of their prosperity, now also should be the time when old scores should be wiped off, and when some sort of readjustment should be made of the iniquitous club rates which, as the result of a senseless downward competition, have come to be accepted as the basis of medical remuneration by many general practitioners. It appears that the changes in wages reported during May affected about 181,200 workpeople, only a few hundred of whom suffered decreases. The net result of all the changes was an average increase of 1s. 0¾d. weekly per head, the principal increase occurring among coal miners, furnace men, and earthenware workers. But if we take the five months from January to May we find that the number of separate individual workpeople who were reported as having received increases or decreases of wages amounted to about 950,500. Of these 944,900 obtained a net average increase of 2s. 2¼d. weekly per head, and 5,600 sustained a net average decrease of 2s. 1½d. weekly per head. Thus the general effect of all the changes was a rise of 2s. 2d. per week in the wages of the 950,500 workpeople affected. Think of it! A rise of £100,750 since Christmas in the amount distributed in wages week by week. Think of the millions of pounds extra now being received in wages by the working classes, and then think of the miserable rates of pay given by clubs and friendly societies for medical attendance, often not exceeding a halfpenny or a penny a week. As working men ourselves—men who work very hard indeed—we naturally are much gratified to hear that "labour" is in such good case. All the same we feel that, under the circumstances, now is the time for some readjustment being made in the standard rates of pay for medical attendance on club patients and friendly societies—rates which always savoured of charity to a far greater extent than was either just or desirable.

**The National Hospital for the Paralysed and Epileptic.**

The difference between the medical staff and the board of management of the National Hospital for the Paralysed and Epileptic, which has been simmering, or shall we not rather say bubbling, for many months, has now boiled over altogether in a public protest, addressed to the governors by the medical staff, in which exception is taken to various points in the management of the institution. The points to which they draw attention are (1) the diet of the patients; (2) the condition, care, and treatment of the patients; (3) the nursing of the hospital; and then they give their opinion as to the cause of what they describe as "the maladministration of the hospital," and detail the manner in which "the present administration has prevented the co-operation of the medical staff and board of management." The extent to which the rupture has grown may be judged by the statement that the signatories to the memorandum feel "compelled to cease communication with the board," and to address the governors directly. In answer to this a circular has been issued stating that steps are being taken to bring the board of management together at the earliest possible date, in order to consider the report of the staff.

**Removal from the Register.**

The lash by which the General Medical Council keeps the profession in order is one which falls with equal weight upon every one whom it hits, and unfortunately seems to hit with great impartiality those who commit great sins and little ones. Nothing short of removing a man's name from the register can the Council do—and nothing greater. Sometimes, after long deliberation, a man's name is removed because he has been found guilty of infamous conduct. Which is surely just. At others, without deliberation or formality, a practitioner's name is erased merely because he has failed to answer a circular sent in a halfpenny wrapper asking his address, which is rather like shooting rabbits with a siege gun. Life is too short for ordinary people to take seriously all the communications shovelled upon them in halfpenny wrappers. There can be no doubt that the proper course to pursue before taking such a serious step as removing a man's name from the register, a step which may arbitrarily and without appeal deprive him of valuable appointments, would be at the least to endeavour by means of a registered letter to find out whether he is still resident at the address given. This is the procedure prescribed in the Dentists' Act, and there seems no reason why the Council should not instruct the Registrar to deal as considerately with medical practitioners as he is compelled by law to do with dentists.

